# KEEPING COOL IN FLORIDA ASSISTED LIVING COMMUNITIES: BARRIERS TO POWER RULE IMPLEMENTATION

**DOI:** 10.1093/geroni/igz038.1239

**Published:** 2019-11-08

**Authors:** Joseph June, Debra J Dobbs, David Dosa, Lindsay J Peterson, Kathryn Hyer

**Affiliations:** 1 School of Aging Studies, University of South Florida, Tampa, Florida, United States; 2 University of South Florida, Tampa, Florida, United States; 3 Brown University School of Public Health, Providence, Rhode Island, United States; 4 University of South Florida School of Aging Studies, Tampa, Florida, United States

## Abstract

In 2018, in response to deaths of nursing home residents after Hurricane Irma, Governor Scott and the Florida legislature enacted an emergency power rule for nursing homes and assisted living communities (ALCs). It mandates these settings to have a generator to regulate ambient air temperatures during loss of primary power by Jan. 1, 2019. However, due to cost and supply challenges the implementation of these plans has been difficult, particularly for small and independently owned ALCs. The purpose of this mixed methods study is to determine the characteristics of ALCs that were able to comply with the rule and concerns raised by ALC administrators. Using data from state regulatory agencies on all Florida ALCs (N=3082), we determine associations between ALC characteristics (size, specialty license, low-income residents) and non-compliance to the rule, using chi-square and t-tests. Additionally, we conducted interviews and focus groups with ALC administrators (N=60) about issues of implementing their emergency power plan. A content analysis approach was used and Atlas.ti v7 was used for initial and axial coding. Some prevalent themes were issues with time frames, coordination between local and state regulations, and financial burden. Themes varied by size and organizational structure of ALCs. Results will inform policy-makers on the barriers faced by ALCs to implement a new regulation that may cause financial difficulties and compromise quality care. This study has implications related to disaster preparedness regulations and their effects on independent ALCs with fewer financial resources.

